# Why Is There No Cure for Tinnitus?

**DOI:** 10.3389/fnins.2019.00802

**Published:** 2019-08-06

**Authors:** Don J. McFerran, David Stockdale, Ralph Holme, Charles H. Large, David M Baguley

**Affiliations:** ^1^Colchester General Hospital, East Suffolk and North Essex NHS Foundation Trust, Colchester, United Kingdom; ^2^British Tinnitus Association, Sheffield, United Kingdom; ^3^Action on Hearing Loss, London, United Kingdom; ^4^Autifony Therapeutics Limited, Stevenage Bioscience Catalyst, Stevenage, United Kingdom; ^5^National Institute for Health Research (NIHR) Nottingham Biomedical Research Centre, Nottingham, United Kingdom; ^6^Hearing Sciences, Division of Clinical Neurosciences, School of Medicine, University of Nottingham, Nottingham, United Kingdom; ^7^Nottingham Audiology Services, Nottingham University Hospitals NHS Trust, Nottingham, United Kingdom

**Keywords:** tinnitus, cure, biomarker, outcome measure, clinical trial design

## Abstract

Tinnitus is unusual for such a common symptom in that there are few treatment options and those that are available are aimed at reducing the impact rather than specifically addressing the tinnitus percept. In particular, there is no drug recommended specifically for the management of tinnitus. Whilst some of the currently available interventions are effective at improving quality of life and reducing tinnitus-associated psychological distress, most show little if any effect on the primary symptom of subjective tinnitus loudness. Studies of the delivery of tinnitus services have demonstrated considerable end-user dissatisfaction and a marked disconnect between the aims of healthcare providers and those of tinnitus patients: patients want their tinnitus loudness reduced and would prefer a pharmacological solution over other modalities. Several studies have shown that tinnitus confers a significant financial burden on healthcare systems and an even greater economic impact on society as a whole. Market research has demonstrated a strong commercial opportunity for an effective pharmacological treatment for tinnitus, but the amount of tinnitus research and financial investment is small compared to other chronic health conditions. There is no single reason for this situation, but rather a series of impediments: tinnitus prevalence is unclear with published figures varying from 5.1 to 42.7%; there is a lack of a clear tinnitus definition and there are multiple subtypes of tinnitus, potentially requiring different treatments; there is a dearth of biomarkers and objective measures for tinnitus; treatment research is associated with a very large placebo effect; the pathophysiology of tinnitus is unclear; animal models are available but research in animals frequently fails to correlate with human studies; there is no clear definition of what constitutes meaningful change or “cure”; the pharmaceutical industry cannot see a clear pathway to distribute their products as many tinnitus clinicians are non-prescribing audiologists. To try and clarify this situation, highlight important areas for research and prevent wasteful duplication of effort, the British Tinnitus Association (BTA) has developed a Map of Tinnitus. This is a repository of evidence-based tinnitus knowledge, designed to be free to access, intuitive, easy to use, adaptable and expandable.

## Introduction

Tinnitus is a common symptom, yet there are few effective treatment options and those that are available are aimed at ameliorating the impact rather than offering hope of a cure. Tinnitus support agencies often reflect their members’ exasperation at the lack of potentially curative treatment options. For example, the online support organization, TinnitusHub^1^, has the following text as part of its Statement of Research: “One thing that both our users and we share in common is a strong desire for a cure. The patient population in general feels frustration and impatience; why isn’t there a cure, why don’t we understand more, why are we not hearing of breakthroughs and feeling hope, where is the funding?” The reasons behind this apparent impasse are complex.

## Patient and Provider Perspectives on Tinnitus Treatment Options

### How Effective Are Current Tinnitus Management Strategies?

Tinnitus management options that have been subjected to randomized controlled trial (RCT) investigation include pharmacological interventions, sound-based interventions, psychological interventions, magnetic stimulation, electrical stimulation, manual physical therapy, relaxation therapy, complementary and alternative medicine (CAM) therapies, education and information, self-help interventions and complex interventions (defined as a combination or two or more of the preceding modalities). Most trials have investigated methods of reducing the day-to-day impact of tinnitus rather than looking for long-term or potentially curative treatments that target the underlying causes of the disorder.

Psychology-based interventions, particularly those based on cognitive behavior therapy (CBT) are often cited as the most efficacious of current tinnitus treatments. Yet this modality is aimed at reducing tinnitus-associated distress rather than reducing the tinnitus *per se*. Systematic review and meta-analysis of trials of CBT for tinnitus have been conducted and have shown that CBT is effective at improving quality of life and reducing tinnitus-associated depression (Martinez-Devesa et al., 2010). However, when the primary outcome of subjective tinnitus loudness is considered, the same systematic review found no evidence of a difference between CBT and either no treatment or another intervention.

### Are Tinnitus Patients Satisfied With Current Tinnitus Services?

For such a common complaint, remarkably little research has been conducted into the aspirations and expectations of patients with tinnitus. Various studies have collected data on perceptions of tinnitus services from general practitioners (GPs), ENT physicians and audiologists (El-Shunnar et al., 2011; Gander et al., 2011; Hoare et al., 2012) but patients’ voices have largely been ignored. A recent study from the United States has redressed this by circulating questionnaires to a group of audiologists and a group of patients (Husain et al., 2018). Data from 230 adults with tinnitus and 68 audiologists were analyzed and revealed a large disconnect between the aspirations of the two communities. When asked to define treatment success, audiologists identifying decreased awareness (77%) and stress/anxiety relief (63%) whereas patients sought reduction of tinnitus loudness (63%), and elimination of tinnitus (57%). The area of greatest agreement was that both groups felt that supplying more information regarding tinnitus is helpful. When patients were asked “how effectively is your healthcare provider able to treat or manage your tinnitus?” 82.6% of respondents replied, “not at all effectively” or “not very effectively.” Only 3.5% thought that their tinnitus had been managed “very effectively” or “extremely effectively.”

A study of tinnitus management in the United Kingdom (McFerran et al., 2018) obtained responses from 937 individuals and demonstrated that the United Kingdom healthcare system performs well at investigating people with tinnitus and excluding serious underlying pathology but markedly less so when it comes to treating the problem: 67.7% of patients who were investigated appropriately were not offered any therapeutic assistance. Many of these expressed their dissatisfaction by returning to their primary care physician only to be rereferred back to secondary care, creating unsatisfactory and expensive revolving door healthcare.

A study of quality of life in patients with tinnitus in Sweden (Zarenoe and Ledin, 2014) included questions regarding the participants’ satisfaction with the healthcare services that they had received for their tinnitus. Out of the total of 376 respondents who commented on treatment given within the ENT clinic, 147 (39.1%) felt they had received “good” or “very good” treatment, 54 (14.4%) felt the treatment was “OK” but 175 (46.5%) felt their treatment was “not good” or they had received no treatment. An optional free text box was included in the questionnaire asking participants about their perception of the tinnitus service they received. Of the 159 answers to this question, 25 respondents commented that they had received an audiometric examination but no treatment.

Dedicated tinnitus services fare better but even here there is dissatisfaction: a survey of patients attending a specialist tinnitus clinic (the Tinnitus Clinic of the Welsh Hearing Institute) were surveyed regarding the perceived benefits and shortcomings of the clinic (Sanchez and Stephens, 2000). The biggest criticism of the clinic was that 17.1% described the interventions and treatments as ineffective.

A research priority setting exercise was undertaken for tinnitus (Hall et al., 2013) in which patients, families, and clinicians partnered to identify the top ten tinnitus research priorities. Of these ten priorities, seven pertained directly to the improvement of existing treatments/therapies, or the identification of novel interventions.

### What Treatment(s) Do Tinnitus Patients Want?

One key question is whether patients with tinnitus would be willing to accept novel treatment modalities for their tinnitus, such as drug treatments or surgery. A study undertaken by Tyler (2012) investigated patient preferences and their willingness to accept and pay for various forms of treatment. The potential treatment modalities comprised external devices, a pill, a cochlear implant, devices surgically implanted onto the surface of the brain or devices surgically implanted into the substance of the brain. This study demonstrated that the most commonly desired treatment modality for tinnitus was an effective drug: 52% would be very likely to try medication if it offered tinnitus loudness and annoyance reduction of a half, rising to 62% if it offered the chance of complete elimination of the percept.

### Is There Financial Benefit to Obtaining a Cure for Tinnitus?

One reason that new treatment options, including pharmaceuticals, have not emerged could be that there would be little financial benefit to companies producing these treatments or little benefit to the healthcare systems and society as a whole. However, this is clearly not the case: the management of tinnitus carries a significant financial burden to healthcare systems and society. Economic modeling of costs in the United Kingdom suggested that the average cost of tinnitus treatment per patient per year in 2016 was GB£717, equating to a total healthcare bill of GB£750 million per annum or approximately 0.6% of the annual healthcare budget (Stockdale et al., 2017). Using previously described methodology (Cima et al., 2012) societal costs were estimated at GB£2.7 billion per annum.

An economic study undertaken in the United States in 2015 gave broadly similar figures to the United Kingdom study, estimating healthcare costs at US$660 per patient per year (Goldstein et al., 2015). A Dutch study (Maes et al., 2013) suggested even higher figures with an estimated mean annual tinnitus-related cost per patient of €1544, though this study made the assumption that all patients with tinnitus were actively seeking help for their condition and may therefore be an overestimate.

Further evidence for the financial and healthcare resource cost of tinnitus is demonstrated by the fact that tinnitus is now the number one service-related disability seen amongst military veterans in the United States. A statement from the foremost American non-profit organization committed to curing tinnitus, The American Tinnitus Association (ATA)^2^, reported that there were 971,990 Veteran’s Administration claims for tinnitus in 2012, resulting in payment of $1.2 billion on tinnitus-related compensation to veterans.

The commercial rewards for a company that could bring an effective pharmacological treatment for tinnitus to market are likely to be considerable. An estimate produced by the United Kingdom hearing charity, RNID (now Action on Hearing Loss^3^) suggested that a novel tinnitus drug could have a product value of $689 million in its first year of launch (Vio and Holme, 2005). This study also estimated that at that time, there were 13 million people in Western Europe and United States actively seeking help for their tinnitus and that 4 million off-label prescriptions for tinnitus were written each year.

### Is the Amount of Ongoing Tinnitus Research Proportionate to the Size of the Problem?

One way of trying to assess the level of research interest in tinnitus is to benchmark it against a range of other neurological or neuropsychiatric disorders. A search of the United States National Library of Medicine trials registration website, clinicaltrials.gov^4^, was conducted using the following parameters:

1. Condition.
2. Interventional studies (clinical trials).
3. Targeted search. Intervention/treatment “drug”.

The conditions entered into the search were chronic pain, depression, anxiety, hearing loss or deafness and tinnitus: results are presented in Table 1.

**TABLE 1:** A list of trials registered on the clinicaltrials.gov website, showing both total number of trials and those trials investigating drug interventions for the relevant condition (such conducted on).

**Condition**	**Total registered trials**	**Interventional trials using drugs**
Chronic pain	2235	855
Depression	5509	2440
Anxiety	2826	921
Hearing loss/deafness	494	94
Tinnitus	200	55

**FIGURE 1:**
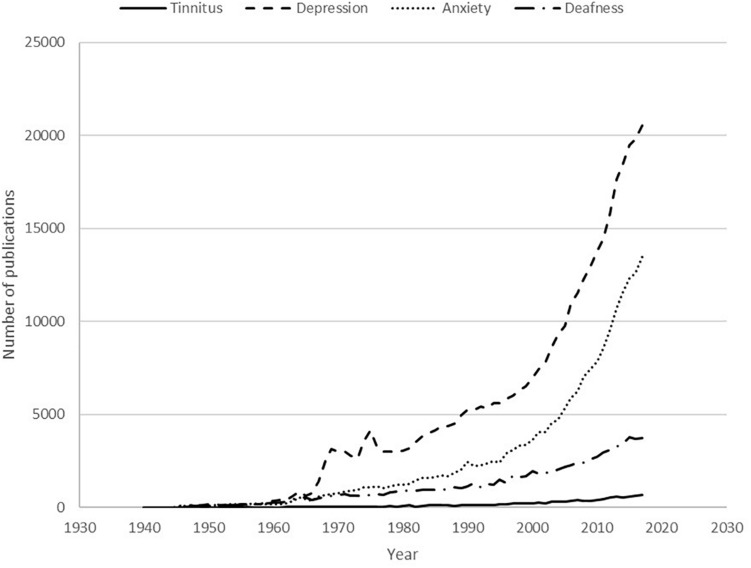
Publications listed on the United States National Library of Medicine PubMed database for the conditions tinnitus, deafness, anxiety or depression in the period from 1940 to 2017.

This is clearly not a systematically accurate reflection of the research interest in these conditions: not all clinical trials are registered on the clinicaltrials.gov website and the website’s search tools detect broad categories but lack precision. Furthermore, many drug trials investigate factors such as drug side effects and safety rather than drug efficacy. Nevertheless, the figures can be used to demonstrate the relative research interest and show that tinnitus falls behind other comparable conditions: depression has over 27 times more registered trials in general and more than 44 times more registered trials relating to drug interventions than tinnitus. The situation is very similar when scientific publications on these conditions are considered. A search of the United States National Library of Medicine’s PubMed^5^ database was performed for the following conditions: depression, anxiety, deafness OR hearing loss, tinnitus. The number of publications per year between 1940 and 2017 is depicted in Figure 1.

**FIGURE 2:**
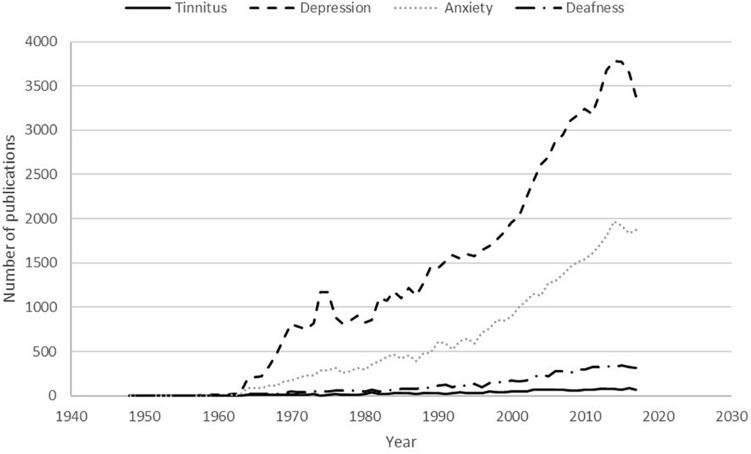
Publications listed on the United States National Library of Medicine PubMed database for the search [(pharmacological treatment) OR (drug treatment)] AND condition (conditions: tinnitus, deafness, anxiety or depression) in the time period from 1940 to 2017.

The search was then repeated for these conditions AND [(pharmacological treatment) OR (drug treatment)]. The results of this search are displayed in graphical form in Figure 2.

The results demonstrate the large difference in research output between the various conditions, with depression having 30.5 times more scientific publications in 2017 compared to tinnitus. When attention is turned to drug therapies, the comparison is even more stark: there were 49.0 times more drug treatment related publications for depression in 2017 compared to the output for tinnitus.

## Obstacles to a Cure

### Supply Versus Demand: A Therapeutic Paradox

The preceding observations clearly demonstrate that there are economic and patient driven pressures to find a cure or at least an effective management paradigm for tinnitus that are not being met by current research resources. In the following sections, we review some of the obstacles that impede the identification and development of new treatment options for patients with tinnitus.

### Tinnitus Research Funding

Cederroth et al. (2013) compared national funding available for hearing and tinnitus research against national funding for diabetes research: in United States, between 2009 and 2011, average annual funding for diabetes by the National Institutes of Health (NIH)^6^ was $913 million compared to $214 million for hearing disorders, of which only $5 million was allocated to tinnitus projects. In the same period in the European Union, funding by the Framework Programme 7 (FP7)^7^ system resulted in annual funding of approximately $60 million for diabetes, compared to $3.3 million for hearing disorder projects. There was no tinnitus research funded by FP7. The editorial recognized that tinnitus research is also funded by charities, other non-governmental organizations and philanthropists but concluded that tinnitus research funding is sparse in comparison with other disorders with similar healthcare burdens.

A more recent study from Blustein (2019) confirmed that NIH funding allocated to hearing loss still remains low, in spite of recent evidence by the Global Burden of Disease (GBD) study which showed that hearing loss is now the 4th leading cause of years lived with disability (YLDs) (Wilson et al., 2017). Information on tinnitus is not yet available in the GBD database – inclusion would be useful as a tool for demonstrating need to both research funders and healthcare organizations.

In contrast, funding in Europe has improved lately with the European Commission awarding financial support in the form of Marie Skłodowska-Curie Actions (MSCA)^8^ grants financed through the Horizon 2020 funding programme^9^. Projects funded through this system include the TIN-ACT: Tinnitus Assessment, Causes and Treatments project^10^ and the European School for Interdisciplinary Tinnitus Research (ESIT) project^11^ (Schlee et al., 2018) which received approximately €3.9 million and €3.8 million respectively. While such funding improvements are very welcome, it is noteworthy that tinnitus projects continue to receive a small proportion of the total funding budget: MCSA grants have been allocated to 405 projects related to diabetes, 75 related to deafness or hearing loss but only nine related to tinnitus.

### Tinnitus Research Structure

Tinnitus research is by nature multidisciplinary and can encompass multiple academic disciplines including auditory neuroscience, psychology, audiology, physiology, pharmacology, computer modeling, bioengineering, and clinical medicine – including both otological surgery and neurosurgery. Globally there are very few research centers where cross-specialty expertise is available to cover and integrate this huge breadth of research topics. Ultimately it may be necessary to review and revise the structure of academic careers in tinnitus. Projects such as ESIT which are training tomorrow’s tinnitus researchers go some way to addressing this deficiency, but more is needed.

### Unclear Tinnitus Prevalence

One of the first issues regarding engagement of the pharmaceutical industry in a search for a tinnitus drug is a lack of agreement on the size of the patient population. A systematic review of tinnitus prevalence studies in adults identified 39 different studies (McCormack et al., 2016). Overall prevalence varied over eightfold from 5.1 to 42.7%. The authors attributed a significant part of this variation to the way in which tinnitus had been defined in the individual studies, but even when the review was restricted to the 12 studies that had used the same definition of tinnitus, prevalence estimates varied almost threefold from 11.9 to 30.3%. When study quality was assessed, almost half the included studies had a high risk of bias and the authors concluded that the data were too heterogeneous to warrant meta-analysis. Furthermore, these prevalence studies do not always explore the impact of the reported tinnitus and hence do not estimate the proportion that would seek pharmaceutical treatment if it became available.

Some studies have attempted to address the size of the disease burden: figures from the United Kingdom, suggest that around 6 million people (10% of the population) have some form of tinnitus, with about 600,000 (1%) experiencing it to an extent that it affects their quality of life (National Institute for Health and Care Excellence, NICE^12^). Another analysis in the United Kingdom by Davis and El Rafaie (2000) from the MRC longitudinal study of hearing of 48,313 people found that 10.1% of adults had experienced episodes of tinnitus lasting more than 5 min, and in 5% the tinnitus was moderately or severely annoying. However, only 0.5% of the study population were affected severely enough for their tinnitus to have a serious impact on their ability to lead a normal life. Clearly the number of people with the symptom and its effect on them are only part of the story: other factors such as the safety and side effect profile of any tinnitus drug and its cost would have to be taken into consideration. However, even limiting take-up to the 0.5–1% of the population with life-altering tinnitus results in a substantial market.

### Ambiguous Tinnitus Definitions and Subtyping

Multiple definitions of tinnitus have been published from “ringing or buzzing in the ears” (Oxford Dictionary^13^) to “the conscious experience of a sound that originates in the head of its owner” (McFadden, 1982) or “the conscious perception of an auditory sensation in the absence of a corresponding external stimulus” (Baguley et al., 2013). None of these definitions are entirely fit for purpose. Ringing in the ears is clearly too simplistic – awareness of tinnitus does not have to be within the ears and many sounds other than ringing are reported. The other definitions (McFadden, 1982; Baguley et al., 2013) are more accurate descriptors of tinnitus, but would include the auditory hallucinations seen in some forms of psychiatric illness. Also, some examples of pulsatile tinnitus are generated mechanically, for example, by muscular or vascular activity. Similarly, some examples of low frequency noise complaint are responses to genuine low-frequency noise in the person’s environment though others are probably phantom perceptions which would fall underneath the tinnitus umbrella (Baguley et al., 2016).

**FIGURE 3:**
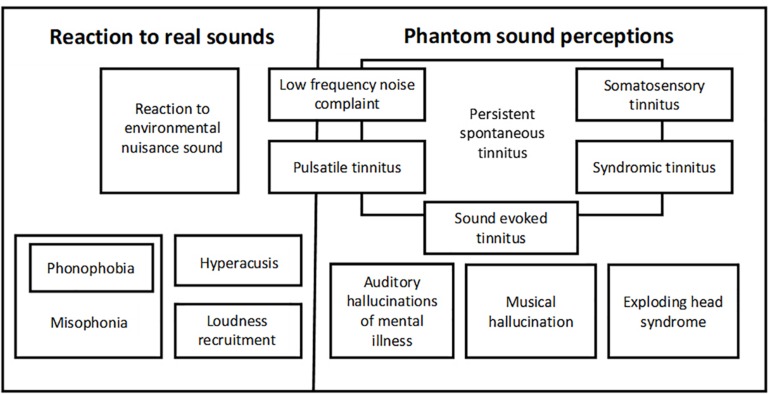
The forms of negative reaction to real and phantom sounds.

The various subdivisions of negative reaction to both real and phantom sounds are depicted graphically in Figure 3.

Most tinnitus trials are conducted in subjects with persistent spontaneous tinnitus also known as subjective idiopathic tinnitus. This is the group of tinnitus sufferers whose tinnitus is non-pulsatile and not related to a small number of specific medical conditions or syndromes including Meniere’s disease, otosclerosis and vestibular schwannoma. However, this group is extremely unlikely to be a homogeneous population either in terms of their tinnitus pathogenesis or tinnitus experience. Describing tinnitus as idiopathic in particular seems inappropriate: the majority of subjects presenting with tinnitus have a hearing loss measurable with conventional pure tone audiometry (Sanchez et al., 2005; Mazurek et al., 2010). There are some patients, perhaps up to one in 10, who have tinnitus in association with normal pure tone audiometry. However, when more sophisticated investigations of cochlear function such as high frequency audiometry (Vielsmeier et al., 2015) or threshold equalizing in noise (TEN) testing (Weisz et al., 2006) are undertaken, most if not all are found to have defects of peripheral auditory function. It is often taken for granted that one form of sensorineural hearing loss is much the same as another, but is this really correct? Is tinnitus arising in someone with noise induced hearing loss identical to the tinnitus in someone whose sensorineural hearing loss is classified as age-related hearing loss, ototoxic medication induced hearing loss or post-head injury hearing loss? Without fully understanding the pathophysiology of different forms of sensorineural hearing loss and its relationship to tinnitus, researchers may well be undertaking studies on heterogenous patient populations that have different underlying mechanisms. This runs a significant risk that subtle treatment effects for specific groups may be missed in the overall picture. Thus, it is possible that effective treatments for some forms of tinnitus already exist, but this effect has been overlooked because results for multiple subtypes have been analyzed as a single group. An interesting example of tinnitus research where test-subject heterogeneity may have affected trial outcome is the story of gabapentin. As its name suggests, gabapentin was initially thought to be a GABA receptor agonist but is now recognized to have its effect by acting on a subsection of voltage-gated calcium channels. It is marketed as an anti-epileptic drug and is also used in the management of certain types of pain. It has been explored in both human and animal studies for possible use in tinnitus. The animal study (Bauer and Brozoski, 2001) suggested that gabapentin was effective at attenuating tinnitus secondary to noise exposure. A subsequent single blind human study undertaken by the same team (Bauer and Brozoski, 2006) suggested that gabapentin was also effective in humans, particularly those whose tinnitus etiology was associated with acoustic trauma. A double-blind trial also reported that gabapentin was effective in treating tinnitus secondary to acoustic trauma (Goljanian Tabrizi et al., 2017). Several other studies, however, have not found gabapentin to be effective (Piccirillo et al., 2007; Witsell et al., 2007; Dehkordi et al., 2011). Only one of these studies divided their participants into those who had experienced significant noise exposure and those who had not (Dehkordi et al., 2011). A history of noise exposure did not affect outcome in this study, though the number of participants who reported sound exposure was low: 16 reported being in noisy environments and 6 reported exposure to explosions. With this conflicting evidence, a large study with robust etiological subtyping of participants would seem the logical next step.

### Clinical Trial Populations

A significant impediment to pharmaceutical industry engagement in the development of new treatments for tinnitus is the perception that, even when some evidence of efficacy is observed in initial efficacy trials (Phase Ib or II), efficacy seems to vanish once the treatment moves into Phase III where the trial populations are more heterogeneous and representative of the intended population once the drug is approved. Participants with tinnitus who are enrolled into initial Phase Ib or II trials often tend to be a discrete subset of the general clinical population of tinnitus sufferers. For example, early Phase Ib or II trial protocols may favor recent onset rather than chronic tinnitus. This may be for mechanism-related reasons: for example, a recent drug development programme for the experimental drug, AM-101, which targets the N-methyl-D-aspartate (NMDA) subtype of glutamate receptors in the cochlea, was predicated on the hypothesis that noise-induced tinnitus arises following damage within the cochlea and might be prevented by early intervention within a few months of the onset of tinnitus (Staecker et al., 2015). There is also a perception that subjective tinnitus may be easier to treat early on, before some of the psychological sequelae are established. Thus, these early efficacy trials will try to give the drug the best chance of exhibiting efficacy before moving into broader populations of tinnitus sufferers. Similarly, early efficacy trials often have an upper age limit of 65 years, which may be due to limits on what may be known about the safety of a new drug in the elderly at this stage of its clinical development, however, since tinnitus increases in prevalence and severity with age, elderly subjects will need to be included in larger Phase III studies. Subjects with very high and catastrophized scores on self-report questionnaires, and/or significant psychiatric impact such as anxiety or depression are also frequently excluded from early trials, despite this being a clinical scenario with very high unmet need.

Another factor influencing early clinical trial design is the evidence that there is a poor correlation between tinnitus loudness and subjective suffering (Meikle et al., 1984). Consequently, drug developers are often keen to dissociate the two in early clinical trials. For example, in order to demonstrate that a new drug targeting the pathophysiology of tinnitus within the auditory system can reduce the tinnitus percept, it may be important to focus on subjects with a consistent tinnitus, but who have limited psychological sequelae associated with the tinnitus.

Robust efficacy is a must in early Phase II trials before drug developers are likely to invest in the much larger Phase III programmes, and thus it is likely that careful choice of inclusion/exclusion criteria will continue. However, the choice of these criteria needs to be better informed by a greater understanding of the heterogeneity of tinnitus pathology, clinical course, and demographic influences. Furthermore, it may be important to conduct additional Phase II studies in diverse populations (e.g., a specific study in the elderly or in subjects with chronic tinnitus) before moving into Phase III. Such expedient drug development strategies are essential to render a clinical trial more straightforward to run, but drug developers must not lose sight of the unmet clinical need.

### Clinical Trial Design

Following on from the expedient selection of tinnitus patients for study, clinical trial design for novel therapies for tinnitus is hampered by other important factors:

1. Lack of biomarkers, objective outcome measures and treatment endpoints.
2. Uncertainly about the duration of treatment that may be required to achieve an improvement.
3. A significant placebo effect that may mask treatment effect.

This situation is compounded by the small size of many Phase II tinnitus clinical trials that have been conducted to date. A further shortcoming is that trial design and endpoints have varied considerably, making it difficult to pool or meta-analyze the results across studies. A good example of this trial-design problem is demonstrated by the use of repetitive transcranial magnetic stimulation (rTMS) for tinnitus. Three editorial articles (Ciminelli et al., 2016; Piccirillo, 2016; Mennemeier and George, 2017) make similar points, drawing from work in the field of mental health: the use of rTMS for treatment resistant depression was unclear for a long time because the evidence relied on small trials using heterogenous methodologies. Eventually, large, well-designed, multicentre trials were conducted which demonstrated that rTMS does have a role in depression, for specific patients using specific treatment protocols. All these editorials recommended a similar approach is taken for tinnitus.

### Tinnitus Measures and Biomarkers

As stated in the previous section, one recurring problem with tinnitus research is that there is no objective way of determining whether someone has tinnitus, no objective way of determining the severity of that tinnitus and no objective way of assessing whether treatments improve tinnitus. A recent systematic review examined the work to date on trying to find suitable objective measures of tinnitus (Jackson et al., 2019). The review identified 21 articles, studying objective tests that included blood tests, electrophysiological measures, radiological measures and balance tests. The review concluded that the quality of evidence was generally poor and had failed to identify any reliable or reproducible objective measures of tinnitus. A biomarker can be defined as “a characteristic that can be objectively measured and evaluated as an indicator of normal biological processes, pathogenic processes or pharmacological responses to a therapeutic intervention” (Puntmann, 2009). Although this may seem to be another way of describing an objective measure of tinnitus, there are distinctions: a suitable biomarker for drug effect or relevant neural process may not necessarily be a measure of tinnitus or tinnitus pathology. Various candidates for a tinnitus biomarker have been considered, including otoacoustic emission testing, auditory brainstem responses (ABR), gap-prepulse inhibition to acoustic startle, pupillometry, functional imaging, magnetoencephalography, genetic markers, blood or saliva components and markers of stress. Nothing has yet been shown to offer the necessary specificity and sensitivity to be used as a biomarker in tinnitus treatment. though some studies have seemed tantalizingly close to discovering a biomarker: ABR studies in tinnitus patients have shown increased latency and reduced amplitude of ABR Wave I compared to measurements in non-tinnitus control subjects (Milloy et al., 2017; Bramhall et al., 2019). However, the findings have shown considerable variability and lack of consistency between studies, suggesting that further work in this area is needed. There have also been some interesting preliminary findings in genetic studies of tinnitus patients. A twins study (Maas et al., 2017) found evidence supporting a degree of heritability in certain forms of tinnitus. A Swedish study (Cederroth et al., 2019) attempted to disentangle the relative contributions of genetic and environmental factors in medically diagnosed tinnitus patients by exploring a large cohort of people who had been adopted as children. This study suggested that clinically significant tinnitus is associated with genetic factors, with a heritability of 32% but that there is no association between shared-environment factors. There are, however, other studies that provide conflicting evidence regarding the genetic contribution to developing tinnitus and this is another research area deserving more detailed exploration.

One factor that hampers work into finding biomarkers is that we do not yet have a large database of the non-audiological phenotypes of tinnitus patients: collecting data such as the biochemical, radiological and genetic characteristics of large numbers of tinnitus patients has not been undertaken. Ideally a biobank dedicated to tinnitus patients should be created (Cederroth et al., 2017; Szczepek et al., 2019).

Whilst work using genetics to identify pharmacological targets is in its infancy (Cook et al., 2014; Vona et al., 2017; Morgan et al., 2018), it is reasonable to expect that further knowledge regarding the genetic contribution to clinically significant tinnitus would be of considerable value.

Without suitable objective markers or biomarkers, tinnitus research in humans currently uses a range of audiometric and self-report questionnaire measures to assess tinnitus severity and treatment effect. Multiple such tools are available and there is no consensus regarding optimum datasets for clinical research. This makes subsequent comparison of trials and meta-analysis of data problematic. A recent multinational working group has tried to address this (Hall et al., 2015, 2019a; Fackrell et al., 2017) and has proposed a basic portfolio of tinnitus “domains” that should constitute a core outcome set for different types of tinnitus research (Hall et al., 2018). Whilst this suggestion is laudable, it remains to be seen if the tinnitus research community adopts these recommendations and it does not provide the unequivocal objective measure that the pharmaceutical industry desires.

A further limitation of the current tools for assessing tinnitus impact is the reliability and repeatability of such measures: self-report measures of tinnitus have an associated risk of variability, as they supply a momentary snapshot whereas the experience of tinnitus changes with time and context. One approach to reducing that is to perform Ecological Momentary Assessment (EMA) (Goldberg et al., 2017; Probst et al., 2017), a technique also used in anxiety, stress, and pain trials (Yang et al., 2019). Evidence regarding the utility of EMA in tinnitus trials is emergent at present.

### Tinnitus Pathophysiology

There are multiple proposed theories regarding the underlying cause of tinnitus, but knowledge is sparse. Most consider that tinnitus may be triggered in the peripheral or central auditory systems or even from outside the classical auditory pathways. Most theories also agree that the processes that cause tinnitus to persist and create distress occur in the brain rather than the ear (Baguley, 2006). Suggested mechanisms include reorganization of the brain’s tonotopic map following deafferentation, increased spontaneous neuronal firing within the auditory brainstem and mid-brain, increased neuronal synchrony, failure of inhibitory pathways, maladaptive auditory-somatosensory plasticity or errors of predictive coding (Roberts et al., 2010; Schaette and Kempter, 2012; Noreña and Farley, 2013; Sedley et al., 2016; Wu et al., 2016; Gentil et al., 2019; Hullfish et al., 2019; Sedley, 2019). Although we know much more about the pathophysiology of tinnitus than we did a decade ago, much of our knowledge is based on animal or computer modeling. Knowledge of what is happening in humans is less clear and we do not yet have a way of determining the pathological mechanism in an individual patient.

### Animal Models of Tinnitus

Animal models have become widely used in tinnitus research, particularly research regarding tinnitus pathogenesis and research into pharmaceutical treatment of tinnitus (von der Behrens, 2014; Eggermont and Roberts, 2015). Yet tinnitus research literature has several instances where apparently effective treatments in animal models have failed to work in humans. Memantine is an antagonist of NMDA glutamate receptors, used in some cases of dementia. Experimental evidence suggested that it is effective in treating tinnitus arising in rats after acoustic trauma (Zheng et al., 2012). A randomized, double-blind study in humans, however, showed no significant change in the primary outcome measure relative to placebo (Figueiredo et al., 2008). Esketamine, the S(+) enantiomer of ketamine, is another NMDA glutamate receptor antagonist that has been explored for use in acute tinnitus, administered as an intratympanic injection. Despite promising animal data (Bing et al., 2015) and initially optimistic human work a large-scale human study, TACTT3-Trial^14^ failed to show efficacy. AUT00063 is an experimental drug that acts as a modulator of the Kv3.1 subtype of potassium channels. Animal research suggests that the drug is very effective at reducing hyperactivity in the auditory brainstem after noise exposure in rodents (Anderson et al., 2018; Glait et al., 2018) and hence might be expected to be effective against similarly generated tinnitus in humans. However, a randomized controlled trial of AUT00063 in humans with subjective tinnitus, QUIET-1, was halted because of lack of efficacy (Hall et al., 2019b). This discrepancy between animal models of tinnitus and clinical trials in humans has various possible explanations: firstly, the pathophysiology of tinnitus in humans may be different from that of laboratory animals (and there remain significant questions about whether the animals do experience tinnitus, and whether our methods for detecting the symptom are reasonable). Secondly, where a drug has failed to show efficacy in a human clinical trial after successful animal studies, it is important to be sure that the drug adequately engaged the pharmacological target in humans. The absence of suitable translational biomarkers is a major hurdle to satisfying this requirement. Thirdly, animal studies and human studies measure different things: animal studies generally use either behavioral tests or gap-prepulse inhibition of the acoustic startle reflex (Galazyuk and Hébert, 2015) to define the presence or absence of tinnitus whereas human studies use self-report and quantify the tinnitus using questionnaires, rating scales or psychoacoustic measures such as tinnitus loudness matching. There is currently no translationally valid outcome measure that can be used in both human and animal studies. Fourthly, some animal studies use outcome measures that may not be detecting tinnitus: the QUIET-1 study measured neural hyperactivity in the dorsal cochlear nucleus of hamsters (Glait et al., 2018). Although the authors argue persuasively that this neural hyperactivity is indicative of tinnitus, other explanations are possible, and the finding could represent hyperacusis rather than tinnitus. Finally, animal studies are very often limited to acute dosing with drugs, whereas clinical trials in humans explore efficacy after multiple days or weeks of dosing. It is important to check in animals that the drug effect does not reduce after chronic dosing which might explain why no efficacy is seen in chronic studies in patients.

It is important to observe that there have been studies where animal and human tinnitus research concur, and it would be wrong to dismiss animal research. Examples where there is positive evidence to support the translational value of animal research prior to human trials include bimodal stimulation using either sound and electrical stimulation of the cervical or trigeminal nerves (Marks et al., 2018) or sound and electrical stimulation of the vagus nerve (Engineer et al., 2011; Tyler et al., 2017).

### Right Drug, Wrong Time?

It has long been suggested that tinnitus pathogenesis is a two-stage process: an initial ignition which can be anywhere in the auditory system including the cochlea, followed by a secondary process of promotion which occurs in the central auditory system and maintains the prominence of the percept (Baguley, 2006). Inherent in this hypothesis is the suggestion that there may be different therapeutic targets, depending on the stage of the tinnitus. Thus, cases of tinnitus ignited by damage to the peripheral auditory system, may benefit from drugs aimed at the cochlea, given at or soon after onset of the symptom, whereas established tinnitus may need centrally acting drugs. What is not clear, is the time frame for the change from peripheral to central targets. Guitton et al. (2003) demonstrated in a rat model that an NMDA antagonist, gacyclidine, administered to the cochlea prevented salicylate induced tinnitus when given simultaneously. As discussed above, Bing et al. (2015) produced data in an animal model suggesting that an NMDA antagonist might benefit noise induced tinnitus. In this trial, the drug was administered 2 days after noise trauma. Subsequent human trials such as TACTT3 failed to demonstrate efficacy but included subjects who had developed their tinnitus up to 3 months previously. This topic regarding potential optimal therapeutic windows needs further exploration.

There is also emerging evidence that the auditory system reacts differently to both noxious stimuli and drugs depending on the time of day. Meltser et al. (2014) demonstrated that mice exposed to noise trauma all showed initial evidence of hearing loss. However, those whose noise exposure was during the daytime recovered within 2 weeks whereas those whose noise exposure was at night developed permanent hearing loss. By experimentally activating tropomyosin receptor kinase type B (TrkB) using a selective agonist, 7,8-dihydroxyflavone (DHF), the mice could be protected against nocturnally induced hearing loss; DHF made no difference to the temporary hearing loss produced by diurnal noise exposure. The relevance of these findings to humans, to tinnitus and to human drug administration remains to be established but EMA measurements have demonstrated that tinnitus loudness varies throughout the day (Probst et al., 2017), suggesting that tinnitus may also be under the influence of circadian factors.

### Setting Treatment Goals

Partly because of a lack of suitable outcome measures, defining what constitutes a cure is problematic. Various studies have looked at the existing tinnitus questionnaire tools and estimated a value for minimum meaningful change (Zeman et al., 2011; Adamchic et al., 2012; Fackrell et al., 2016) but this represents the smallest change above the measurement error and clinically this is improvement rather than cure. Similarly, use of tinnitus loudness estimates is flawed because of poor sensitivity and large measurement error (Hall et al., 2017). It is likely that tinnitus patients would suggest that the answer to defining a cure is simple: complete eradication of the tinnitus percept. However, several studies (Heller and Bergman, 1953; Del Bo et al., 2008) have shown that people with normal ears, normal hearing and no tinnitus become aware of phantom auditory perceptions when they are placed in soundproof environments. It may therefore be unrealistic to set total eradication of tinnitus percept as the primary goal and more work is needed to ascertain what healthcare providers, purchasers and patients will accept as a clinically meaningful improvement in order to guide clinical trial design. Although sounding less positive, it may be preferable to use different terminology and set the goal as remission from tinnitus rather than the more semantically confusing concept of “cure.”

### Healthcare Organization

Other potential hurdles to attracting pharmaceutical research interest are the lack of a clear route to market with no established regulatory pathway and the lack of a precedent for pricing and reimbursement of a tinnitus drug. Another issue that is particularly relevant to the American healthcare market is the lack of a suitable healthcare structure for tinnitus patients – most patients in the United States currently see audiologists, who cannot prescribe medication. Clearly all of this might change if a promising drug therapy were to make its way through clinical trials.

## Roadmap to a Cure

### Development of Tinnitus Cure Map

The preceding text demonstrates the challenges of tinnitus research. We need more focus on definitions, subtyping and outcome measures; we need research that uses common methodologies, making comparison and meta-analysis easier; we need to ensure that researchers are focussed on what funders and patients want. To try and clarify this complex subject, the British Tinnitus Association (BTA)^15^ has developed a Tinnitus Cure Map. This is an attempt to try and summarize the current tinnitus research, demonstrating knowledge gaps but also demonstrating areas where we already know the answer, blind alleys that do not need further exploration. The aim is also to highlight research opportunities and act as an up to date repository of evidence-based tinnitus knowledge.

The map was developed within the BTA and involved consultation with relevant stakeholder groups, including members of tinnitus patient support groups, BTA members, BTA Professional Advisers Committee members and British Society of Audiology Tinnitus and Hyperacusis Special Interest Group^16^. Criteria were that the map should be free to access, intuitive and easy to use, adaptable and expandable.

**FIGURE 4:**
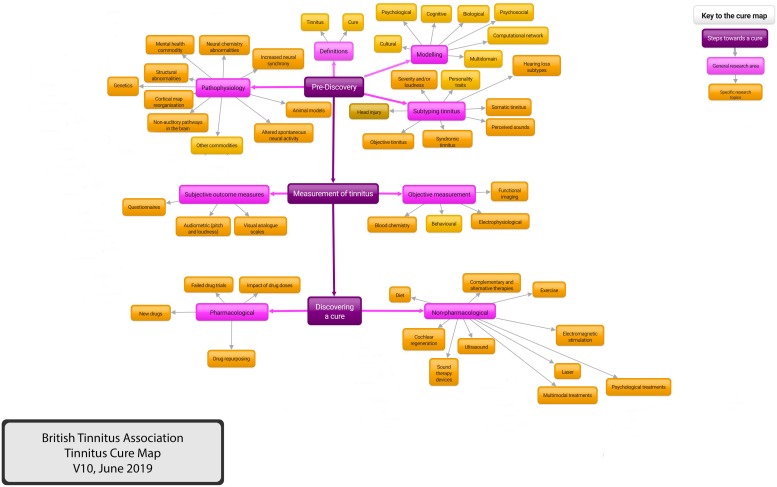
The British Tinnitus Association Tinnitus Cure Map. A representation of tinnitus research areas. An interactive version accessed via the internet is being developed, demonstrating knowledge gaps but also demonstrating areas where we already know the answer and blind alleys that do not need further exploration. The interactive version will connect to other internet resources via hyperlinks. For purposes of clarity, this version of the Cure Map has been limited to three levels. A more comprehensive version with four levels is included in Supplementary Material.

A paper copy of the map has been produced using four heading levels: Steps Toward a Cure, General Research Area, Specific research Area and Individual Projects. A copy of the map limited to three levels for clarity is shown (Figure 4). A version that uses four levels is included in Supplementary Material.

An electronic version is under development. This has no theoretical limit to the number of levels and this version utilizes pop-ups to display detailed content and hyperlinks to external content. The external content is the highest level of evidence available on that topic, using the Oxford Centre for Evidence-based Medicine^17^ criteria.

### The Tinnitus Cure Map as a Lobbying Tool

In addition to providing a comprehensive repository of the current evidence base regarding tinnitus, we hope that the map can be used by charities, other patient groups and individual tinnitus patients to demonstrate to politicians, research funders, the pharmaceutical industry and healthcare organizations the size of the tinnitus problem and the need for a much enhanced research footprint.

## Conclusion

Whilst an encouraging upturn in the volume of tinnitus research being performed is evident, it is also apparent that a step change will be needed to deliver progress toward truly effective treatments. Several building blocks for that need putting in place, including biomarkers, robust outcome measures, and meaningful subtyping of clinical phenotypes. Such work will need to be interdisciplinary and international and will need to engage researchers and clinicians along the whole of the translational research pathway. The role of industry in this endeavor is fundamental, utilizing experience in clinical trial design, and attracting resources for large scale trials that intentionally address the clinical need, incorporating the views of patients/families as well as clinicians and researchers. The opportunities for societal financial benefit and the alleviation of tinnitus related burden and distress are substantial.

## Author Contributions

DM and DS conceived and designed the work. DM helped to produce the Map of Tinnitus and wrote the manuscript. DB, RH, CL, and DS reviewed and edited the manuscript and contributed intellectually to the content. All authors approved the final manuscript for submission.

## Disclaimer

DB is supported by the UK NIHR. DS is an employee of the British Tinnitus Association. DM is a Trustee of the British Tinnitus Association. RH is an employee of Action on Hearing Loss. CL is an employee and shareholder of Autifony Therapeutics Limited. The views expressed herein are their own and may not reflect those of their affiliated organizations.

## Conflict of Interest Statement

The authors declare that the research was conducted in the absence of any commercial or financial relationships that could be construed as a potential conflict of interest.

## Footnotes

1 https://www.tinnitushub.com/

2 https://www.ata.org/news/press-release/treating-and-curing-tinnitus-part-our-national-commitment-veterans

3 https://www.actiononhearingloss.org.uk

4 https://clinicaltrials.gov/

5 https://www.ncbi.nlm.nih.gov/pubmed

6 https://www.nih.gov/

7 https://ec.europa.eu/research/fp7

8 https://ec.europa.eu/programmes/horizon2020/en/h2020-section/marie-sklodowska-curie-actions

9 https://ec.europa.eu/programmes/horizon2020

10 https://tinact.eu/

11 https://esit.tinnitusresearch.net/

12 https://cks.nice.org.uk/tinnitus#!topicSummary

13 https://en.oxforddictionaries.com/definition/tinnitus

14 https://www.epgonline.org/global/news/keyzilen-fails-phase-iii-tactt3-trial-to-treat-tinnitus—auris-medical-.html

15 https://www.tinnitus.org.uk/

16 https://www.thebsa.org.uk/bsa-groups/tinnitus-hyperacusis-special-interest-group/

17 https://www.cebm.net/2016/05/ocebm-levels-of-evidence/

## Supplementary Material

FIGURE S1 The British Tinnitus Association Tinnitus Cure Map. A representation of tinnitus research areas. An interactive version accessed via the internet is being developed, demonstrating knowledge gaps but also demonstrating areas where we already know the answer and blind alleys that do not need further exploration. The interactive version will connect to other internet resources via hyperlinks. This version of the Cure Map uses four levels, which is the maximum number of levels envisaged for the paper version of the map.
