# Supplementary figures and images for: Why Is There No Cure for Tinnitus?
Source: Front Neurosci. 2019 Aug 6;13:802. doi: 10.3389/fnins.2019.00802 (PMC6691100; doi:10.3389/fnins.2019.00802)

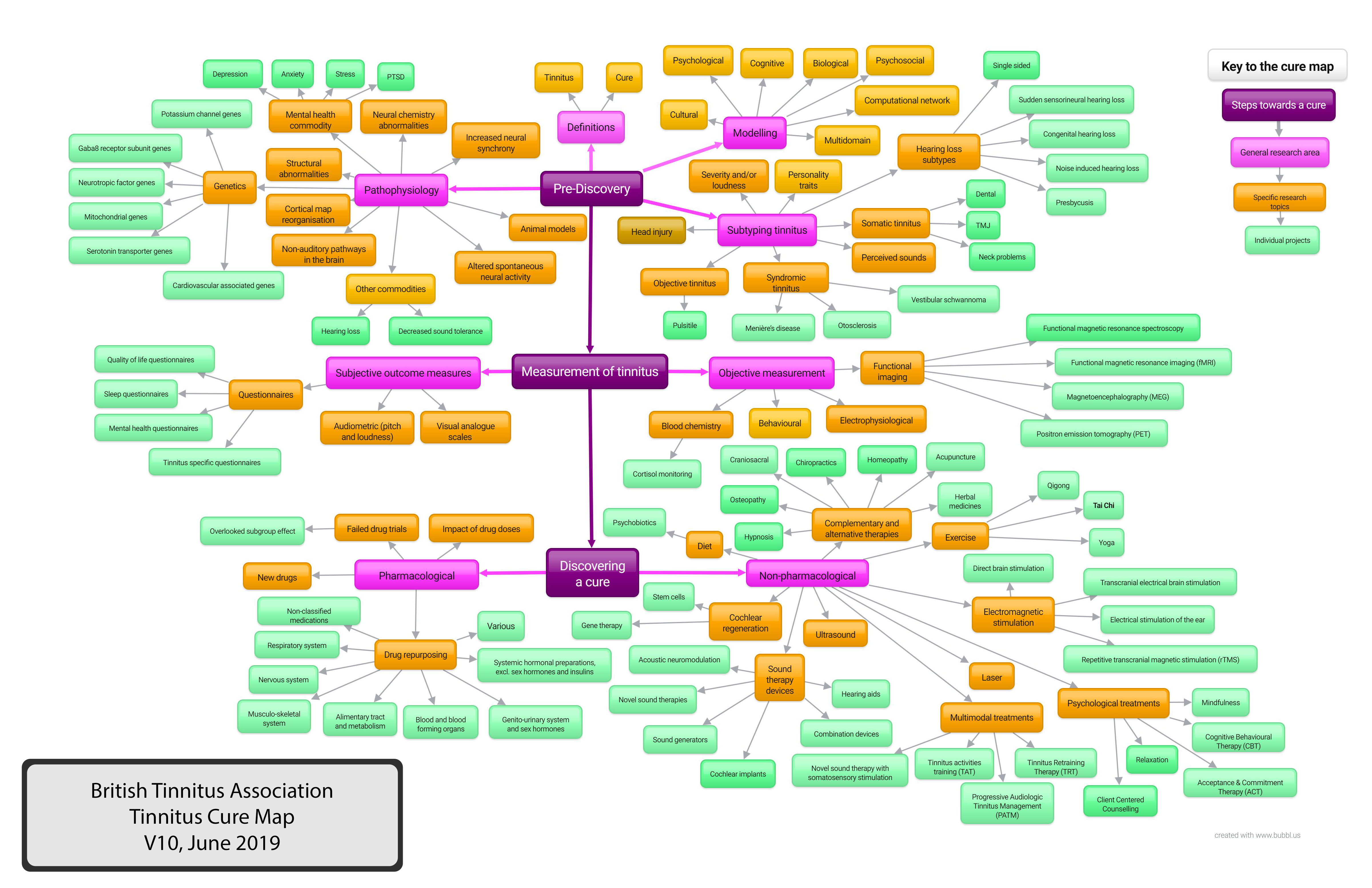

Supplement: FIGURE S1 — The British Tinnitus Association Tinnitus Cure Map. A representation of tinnitus research areas. An interactive version accessed via the internet is being developed, demonstrating knowledge gaps but also demonstrating areas where we already know the answer and blind alleys that do not need further exploration. The interactive version will connect to other internet resources via hyperlinks. This version of the Cure Map uses four levels, which is the maximum number of levels envisaged for the paper version of the map. [file Image_1.JPEG]
